# Research progress on the pathogenesis of Graves’ ophthalmopathy: Based on immunity, noncoding RNA and exosomes

**DOI:** 10.3389/fimmu.2022.952954

**Published:** 2022-08-23

**Authors:** Jingyi Zheng, Honghong Duan, Sufang You, Bo Liang, Yuping Chen, Huibin Huang

**Affiliations:** ^1^ The Second Clinical Medical College of Fujian Medical University, Quanzhou, China; ^2^ Department of Endocrinology, The Second Affiliated Hospital of Fujian Medical University, Quanzhou, China; ^3^ Department of Gynaecology and Obstetrics, The Second Affiliated Hospital of Fujian Medical University, Quanzhou, China

**Keywords:** Graves’ ophthalmopathy, cellular immunity, autoantigen, noncoding RNA, exosomes, biologics

## Abstract

Graves’ ophthalmopathy (GO), also known as thyroid-associated ophthalmopathy, is a common potentially vision-threatening organ-specific autoimmune disease and the most common extrathyroidal manifestation of Graves’ disease. It can happen to those who have hyperthyroidism or euthyroidism. At present, the pathogenesis of GO has not been fully elucidated, and the majority of clinical treatments are symptomatic. Therefore, we are eager to discover any new therapeutic strategies that target the etiology of GO. To provide fresh ideas for the creation of new therapeutic techniques, this study primarily discusses the research state and progress of GO-related pathogenesis from the perspectives of GO’s cellular immunity, autoantigens, non-coding RNAs, and exosomes.

## 1 Introduction

Graves’ ophthalmopathy (GO), also known as thyroid-associated ophthalmopathy, is an organ-specific autoimmune disease that is vision-threatening and cosmetically damaging. GO is an ocular abnormality in Graves’ disease (GD) and occurs in approximately 30% to 50% of GD patients (1). GO can occur before, during or after the onset of thyroid disease. Signs and symptoms of GO include eyelid retraction, diplopia, proptosis, exposure keratitis, corneal clouding and ulceration, and in severe cases, vision-threatening compressive optic neuropathy (2). Corneal scarring caused by exposure or optic nerve compression can result in vision loss or possibly blindness, impacting the patient’s quality of life significantly (3). Because the pathogenesis of GO has not yet been elucidated, there is no cure and treatment is limited, and symptomatic treatment is still the conventional treatment in clinical practice. According to recent research, GO is a multifactorial disease involving cellular immunity, autoantigens, non-coding RNAs, and exosomes. In this paper, we will review the pathogenesis of GO through several of these factors.

## 2 Immune factors

### 2.1 Cellular immunity

**Figure 1:**
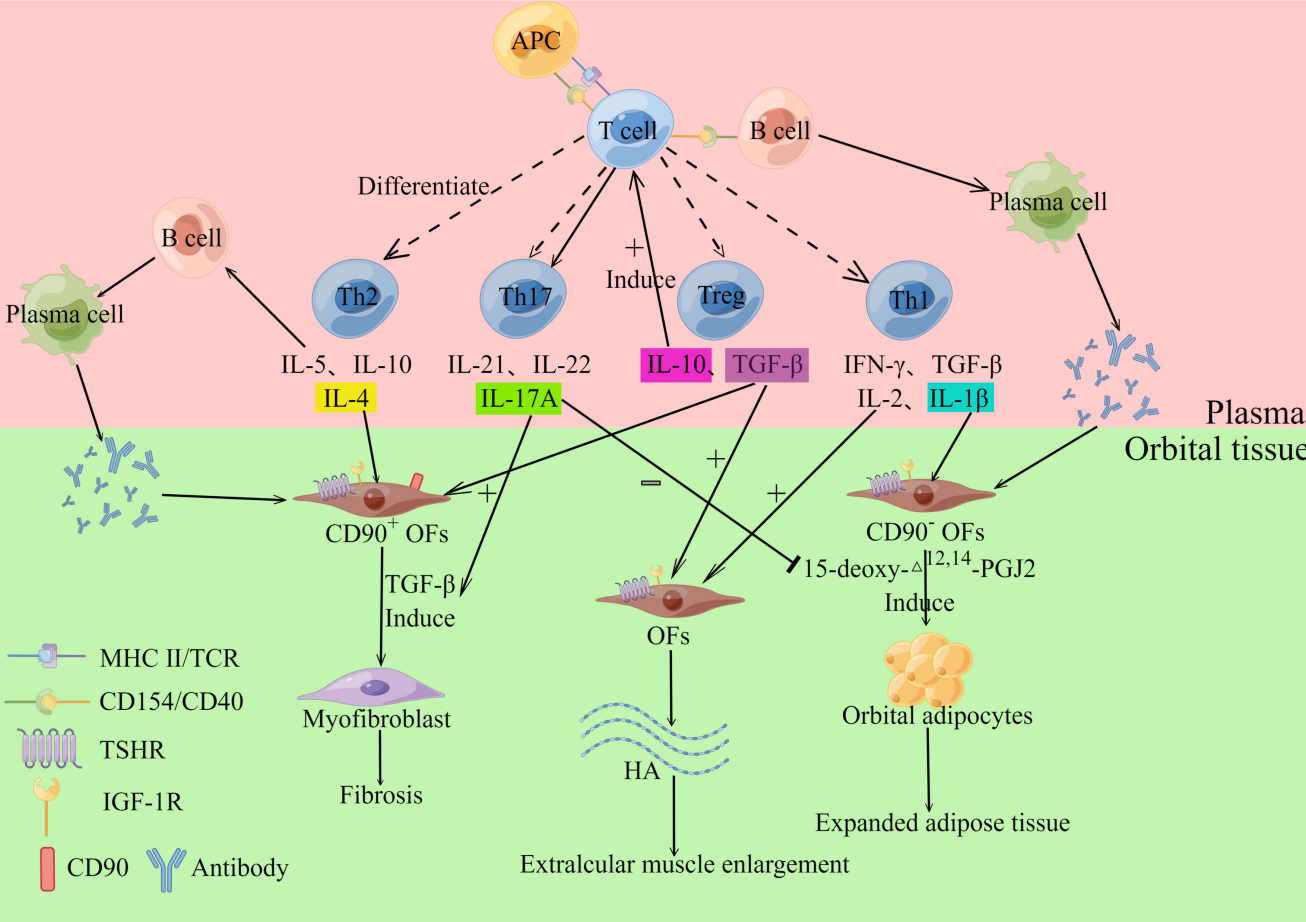
Cellular immunity. T cells can differentiate into Th1, Th2, Th17 and Treg cells, which can secrete various cytokines. Th1 cells can secrete IL-1β, IL-2, IFN-γ and TNF-α, and the above cytokines acting on Orbital fibroblasts (OFs) can induce their synthesis and release of hyaluronan (HA). IL-1β also stimulates OFs to differentiate into adipocytes. Th2 cells release IL-5, IL-10, and IL-4, which stimulate B cells and participate in humoral immunity. IL-4 also stimulates OFs to produce collagen, which participates in the GO fibrosis response. Th17 cells mainly secreted IL-17A, IL-21, and IL-22, among which IL-17A promoted TGF-β-induced fibrosis of CD90^+^ OFs while inhibiting 15-deoxy-△^12,14^-PGJ2-induced adipogenesis of CD90^-^OFs. Treg cells mainly secrete IL-10 and TGF-β. IL-10 induces T cells to differentiate into pathogenic Th17 cells. TGF-β induces OFs to produce HA and induce OFs to differentiate into myofibroblasts. The inflammatory mediator (Il-1β) that promotes adipogenesis activates CD90^-^ OFs to differentiate into adipocytes. In contrast, CD90^+^ orbital fibroblasts were activated by TGF-β and differentiated into myofibroblasts. By Figdraw (www.figdraw.com).

In the orbital tissues and extraocular muscles of GO patients, there are numerous localized and diffuse monocyte infiltrates and mucopolysaccharide deposits, with the infiltrating cells primarily CD4^+^ T lymphocytes, but also modest numbers of neutrophils and plasma cells (2). This shows that the main pathophysiology of GO is cellular immunity (**Figure 1**). CD4^+^ T lymphocytes can be divided into helper T cells1 (Th1 cells), Th2 cells, Th17 cells and regulatory T cells (Treg cells) (4), all of the cells listed above play a crucial part in GO pathogenesis. The target cells involved in GO autoimmunity include: infiltrating auto-reactive T cells and orbital fibroblasts (OFs) located between the extra-orbital muscles (2). T lymphocytes interact with OFs through CD40-CD154 costimulatory pathway (2). When OFs are activated, they release a considerable number of cytokines and extracellular matrix (ECM), causing severe orbital inflammation and tissue remodeling. Also, the heterogeneity of OFs determines the outcome and clinical presentation of orbital tissue remodeling. CD90 can be expressed on the surface of OFs, and CD90^+^ facilitates the fibrosis of OFs, while CD90^-^ tends to cause adipogenesis (5). During the active phase of orbital tissue changes, the volume of tissue around the eye increases due to inflammatory cell infiltration and orbital tissue edema, which in turn results in an increase in intraocular pressure (6), and the eye moves beyond the bony rim of the orbit, leading to exophthalmos. The major pathological changes in GO include cytokine production and inflammation, adipogenesis, hyaluronan synthesis and myofibrillogenesis (6).

According to Aniszewski et al., orbital tissue in patients with less than two years of hyperthyroidism mostly invaded Th1 cells, whereas orbital tissue in patients with more than two years of hyperthyroidism predominantly infiltrated Th2 cells (7). It can be hypothesized that cell-mediated (Th1-type) immune responses predominate in the early phases of GO, whereas humoral immunity (Th2-type) plays a larger role in the latter stages.Th1 cells produce cytokines including interferon (IFN)-γ, interleukin (IL)-1, IL-2, and tumor necrosis factor (TNF)-α, which cause OFs to create and release significant amounts of glycosaminoglycans like hydrophilic hyaluronan, resulting in swelling of orbital tissues (especially the extraocular muscles) (8). Among others, it has also been shown that IL-1β is associated with adipogenesis in GO (9). The association between elevated circulating levels of TNF-α in GO patients and the severity of GO suggests that monoclonal antibodies against TNF-α, such as Infliximab and Etanercept, could be used to treat GO (10). Studies have shown that both of these drugs have a positive effect on the treatment of GO, reducing the inflammatory response to GO and improving ocular symptoms (11, 12). Th2 cells normally produce IL-4, IL-5 and IL-10. These cytokines are mainly involved in humoral immunity and can stimulate the activation of B cells, leading to the production of autoantibodies (13). Similar to IFN-γ, IL-4 stimulates the proliferation of OFs. Unlike IFN-γ, IL-4 stimulates type IV collagen synthesis, and high doses of IL-4 (>1ug/L) inhibit hyaluronan production (14). Since type IV collagen production is associated with fibrosis and high doses of IL-4 inhibit hyaluronan production, we can speculate that high IL-4 production is a marker of late GO and one of the main causes of orbital tissue fibrosis in late GO.

The above orbital inflammatory state combined with the hyperthyroid state allows excessive production of reactive oxygen species (ROS), mainly superoxide anion and hydrogen peroxide. As with many inflammatory diseases, GO is associated with oxidative stress because of its reduced antioxidant capacity to cope with increased ROS production, which consequently leads to oxidative stress (15). Many studies support the pathogenic role of oxidative stress in the pathogenesis of GO, and researchers have detected ROS in the OFs of GO patients (16). Regemorter et al. reported that an increase in ROS in GO may eventually lead to an increase in pro-inflammatory cytokines (e.g. IL-6), resulting in an inflammatory response in GO (15). Kim et al. reported that ROS production was measured during GO adipocyte differentiation and that the increase in ROS levels was maximal on day 1 and then ROS levels remained at approximately 200% of day 0 levels (17). Therefore, we can conclude that ROS can induce GO to generate adipocytes. Bartalena et al. reported that ROS stimulated the proliferation and differentiation of OFs and produced hyaluronan (18). Meanwhile, ROS up-regulated the expression of human leukocyte antigen-DR and heat shock protein-72, which contributed to the process of GO inflammatory response by participating in T-lymphocyte activation. In summary, we can conclude that oxidative stress is involved in the pathogenesis of GO.

The early studies of GO focused on cytokines produced by Th1 and Th2 cells and their pathogenic effects. Recent researches have shown that Th17 cells are also involved in the disease development process of GO. Several studies have shown that in studies examining the relationship between Th17 cells and GO, higher levels of Th17 cells were detected in GO patients compared to healthy controls, especially those with a clinical activity score (CAS) ≥3 (5). Th17 cells mainly secrete IL-17A, IL-21, and IL-22, which can cause tissue inflammation and autoimmune. They also found that IL-17A also promoted transforming growth factor (TGF)-β-induced fibrosis in CD90^+^ OFs while inhibiting 15-deoxy-△^12,14^-PGJ2 induced adipogenesis in CD90^-^ OFs (5). It has been shown that Th17 cells produce high levels of IL-17A acting on OFs, causing OFs to secrete more inflammatory cytokines and ECM and express fibrosis-related proteins, which is one of the causes of the inflammatory response and the development of fibrosis (5). Since then, further insights on the investigation of Th17 cells in GO patients have been presented by researchers. Fang et al. found that most GO patients had CCR6^+^ Th17 cells expressing IL-17A and that IL-17A production by CCR6^+^ Th17 cells gradually decreased from active GO patients to inactive GO patients (19). Meanwhile, the increase in CAS of GO patients was synchronized with the increase in the number of CCR6^+^Th17 cells. This suggests that CCR6^+^ Th17 cells are associated with GO activity and that they may maintain the development of GO orbital inflammation. We can also speculate that CCR6^+^ Th17 cells, similar to Th1 cells, have a role in the early phases of GO. The symptoms of GO can manifest not only in the orbital connective tissue, but also in the lacrimal glands, such as ocular surface inflammation and xerophthalmia (2). Huang et al. found that IL-17A produced by Th17 cells in the lacrimal glands promoted the differentiation of TGF-β-initiated myofibroblasts in GO lacrimal fibroblasts and 15-deoxy-△^12,14^-PGJ2-initiated adipocytes (20). They concluded that the above process could be one of the major reasons for GO lacrimal glands fibrosis. Since IL-17A has similar pro-inflammatory, pro-fibrotic and pro-adipogenic mechanisms in lacrimal and orbital connective tissues, targeting Th17 cells may have therapeutic effects on both tissues. SHR-1314 is a recombinant humanized monoclonal antibody against IL-17A that has been demonstrated to be effective in clinical trials for a variety of autoimmune illnesses (21). Therefore, we speculate that it may also be effective in the treatment of GO, and we can carry out clinical studies on the efficacy and safety of SHR-1314 for the treatment of GO in the future to provide a new approach for the treatment of GO. In recent years, researchers have also proposed the concept of Th17 cell plasticity, which means that Th17 cells can transform into other CD4^+^ T cell subsets, such as Th1 and Th2 cells (22). We envisioned whether pathogenic Th17 cells could be selectively depleted while normal cells were preserved by identifying particular surface markers of pathogenic Th17 cells. Or study the mechanism by which Th17 cells are transformed into other cells, and stop the disease by intervening to transform pathogenic cells into cells that are beneficial to the body.

Treg cells are immunosuppressive T-cell subtypes, and it has been shown that reduced numbers or functional defects of Treg cells are related to the development of various autoimmune diseases (23). However, the mechanism of Treg cells in the development of GO is unclear. The majority of Treg cells are predominantly Foxp3^+^, which mainly produce TGF-β, IL-10 and other anti-inflammatory cytokines, and they can exert their immune effects through a variety of different mechanisms. Hyaluronan is a potential regulatory substance that plays a role in the differentiation of orbital fibroblasts into myofibroblasts induced by TGF-β. It has been shown that TGF-β induces the production of hyaluronan by OFs (24). Ma et al. found that TGF-β1 can induce OFs to differentiate into myofibroblasts *via* HA-CD44 signaling, characterized by α-smooth muscle actin up-regulated (24). The same results were reported by Wang et al. who noted that TGF-β1 stimulation of OFs from GO patients produced higher expression of fibrosis and extracellular matrix production markers (such as α-SMA, FN1 and COL1A1) than stimulation of OFs from GO-free subjects (25). It also indicates that OFs from GO patients have differentiated into myofibroblasts. Wu et al. showed that TGF-β1 can induce the transdifferentiation of OFs into myofibroblasts through the MAPK signaling pathway, characterized by enhanced expression of fibrotic proteins such as α-smooth muscle actin, connective tissue growth factor, and fibronectin (26). In addition, their study showed that TGF-β1 induces the up-regulated of type I collagen with the accumulation of ECM proteins. The fibrosis and the accumulation of ECM together lead to remodeling of GO extraocular muscles. In conclusion, TGF-β plays an important role in orbital tissue fibrosis in GO patients. TGF-β can also promote the proliferation, differentiation and survival of lymphocytes and other immune cells to maintain immune tolerance (27). IL-10 induces the differentiation of T cells into pathogenic Th17 cells (28), and also interferes with the migration of Th1 cells to sites of intestinal inflammation (29). Another study suggests that the number of Treg cells may be related to the severity of the inflammatory response in GO, and that GO with a higher frequency of Treg cells tends to exhibit a decreased clinical course (30). Muñoz A et al. found that CD69^+^ Foxp3^-^ Treg cells were significantly increased in the peripheral blood of GO patients and that there was a positive correlation with GO activity (31). Therefore, the number of Treg cells in the peripheral blood of GO patients can be used as a predictor of the clinical course. Furthermore, in recent years, it has also been reported that dysregulation of Treg/Th17 balance is also involved in the pathogenesis of GO (32). Therefore, we hypothesized that inflammation and fibrosis in GO orbital tissues may be attenuated when the Treg/Th17 ratio is elevated. Conversely, disease symptoms may develop and/or worsen when Treg/Th17 is decreased.

### 2.2 GO-associated autoantigens

#### 2.2.1 TSHR

**Figure 2:**
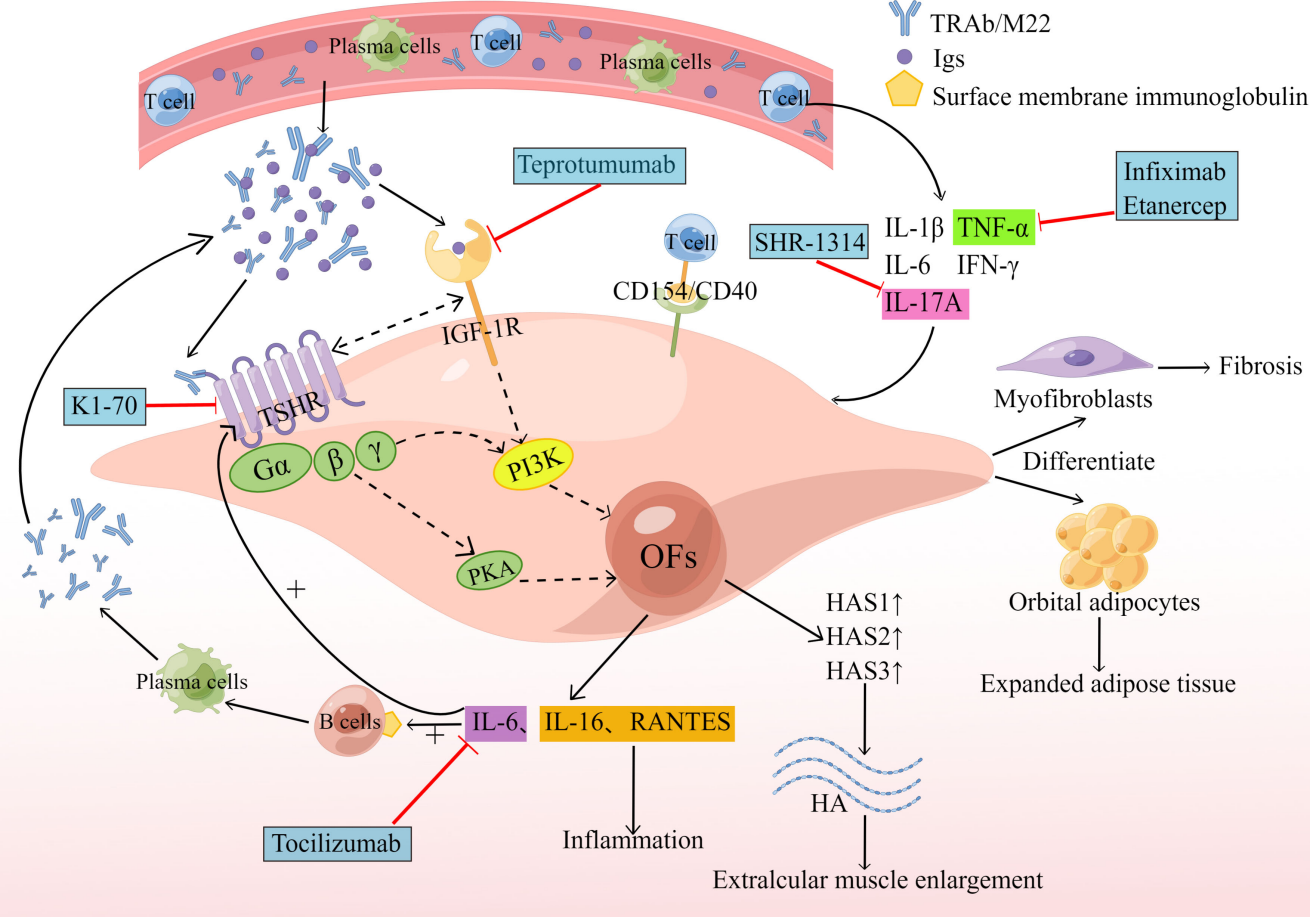
Humoral immunity and biologics. The circulating TRAb and M22 act on TSHR to induce cAMP production, which activates the PI3K cascade reaction and promotes the production of HAS1, HAS2 and HAS3, resulting in increased production of hyaluronan, leading to extraocular muscle hypertrophy. M22 also increases the expression and secretion of IL-6. Subsequently, the increased expression of IL-6 promotes the expression of the autoantigen TSHR by OFs and also drives B-cell immunoglobulin (Ig) production, and plasma cell development. Serum Igs from GO patients bind to IGF-1R and produce IL-16 and RANTES, promoting an inflammatory response. TSHR and IGF-1R can form physical and functional protein complexes that can interact to increase signaling when activated by their respective ligands, resulting in increased hyaluronan (HA) and IL-6 production. The generated HA and IL-6 then repeat the above reaction, aggravating the GO pathological process. New therapeutic drugs are indicated by blue boxes and red lines. By Figdraw (www.figdraw.com).

The thyroid stimulating hormone receptor (TSHR) is a G protein-coupled receptor that is a glycoprotein hormone receptor. It is the major autoantigen of GO and can promote cAMP synthesis and activate the PI3K cascade. In 1976, Kriss et al. first proposed the concept of a common antigen between thyroid and orbital affected tissues, in which TSHR expressed on OFs is a cross-cutting antigen causing autoimmune reactions in GO patients (33). Several studies have confirmed that TSHR mRNA can be detected in the orbital connective tissue of GO patients, and the expression of TSHR in the orbital adipose tissue of GO patients is higher than that of normal tissue (34). Therefore, we can speculate that the activation of TSHR can induce adipogenesis in orbital tissue. The production of new adipose in the eye frame of GO patients also enhances the expression of TSHR in this tissue (6). Lu et al. demonstrated that TSH can stimulate adipogenesis even without adipogenic factors by using the *in vitro* model of adipogenesis of mouse embryonic stem cells (35). GO patients have circulating autoantibodies against TSHR, and M22 is a monoclonal antibody against TSHR. M22 has been shown to increase cAMP production as well as the production of three hyaluronan synthases (HAS1, HAS2 and HAS3), thus activating OFs and increasing hyaluronan production (36). In addition, M22 increases the expression and secretion of IL-6 in pre-adipose fibroblasts and adipocytes of GO patients, while IL-6 promotes the expression of the autoantigen TSHR by OFs, and also drives B-cell immunoglobulin production, plasma cell development (**Figure 2**), aggravating the autoimmune response and influencing the disease’s clinical activity (37). IL-6 is negatively related to GO duration, and positively related to CAS (38). The level of anti-TSHR antibodies relates to the severity and clinical activity of GO. The production of adipose and hyaluronan in OFs reflects the remodeling of GO orbital tissue.

Tocilizumab is a humanized monoclonal antibody against IL-6R, which can block IL-6 activity and signal transduction. Tocilizumab significantly improved disease activity and severity in patients with corticosteroid-resistant GO, and its effects have been shown in a multicenter, randomized, double-blind trial conducted in Spain (39). Human monoclonal autoantibody K1-70 is a particular TSHR antagonist with therapeutic potential for patients who block the effect of thyroid stimulants on TSHR. After administering K1-70 to a female patient with GD, severe GO and follicular thyroid cancer, it was reported that the patient’s CAS and proptosis improved significantly (40). Results from a recent phase I clinical trial of K1-70™ in the treatment of patients with GD and GO showed that K1-70™ was safe and well-tolerated by patients and produced the expected pharmacodynamic effects without immunogenic reactions (41). As a new drug, K1-70™ shows considerable promise for application with the ability to block the effect of thyroid-stimulating agents on TSHR.

#### 2.2.2 IGF-1R

It has recently been shown that the insulin-like growth factor-1 receptor (IGF-1R) is overexpressed in GO (42). We speculate that it may be another important autoantigen of GO. The serum immunoglobulins (Igs) from GO patients act on OFs to produce T-cell chemotactic agents such as IL-16 and chemokine RANTES (43)(**Figure 2**), which regulate the expression and secretion of activated, normal T cells and also facilitate the transport of CD4^+^ T cells (44), allowing for a sustained GO autoimmune response. This response can be weakened by blocking autoantibodies to IGF-1R or by transfecting fibroblasts with dominant-negative mutant IGF-1R (6), suggesting that IGF-1R signaling mediates this process. IGF-1R can also interact with autoantibodies against the receptor and participate in adipogenesis and hyaluronan synthesis (45). However, the effect of IGF-1R on hyaluronan synthesis appears to be indirect, as IGF-1 itself does not increase HAS2 transcription.

As the study progressed, researchers found that IGF-1R can also be involved in GO pathogenesis through the IGF-1R/TSHR crosstalk pathway, where GO Igs activate crosstalk by binding to TSHR on OFs (46). Kriege et al. proposed that IGF-1R and TSHR must be physically adjacent to each other to make their signaling pathways the same, and that arrestin-β-1 can provide a scaffold for the TSHR/IGF-1R protein complex, which provides the basis for crosstalk between the two receptors (47). Thus, TSHR and IGF-1R can form physical and functional protein complexes. It has been shown that IGF-1R and TSHR can interact to increase signaling when activated by their respective ligands, and that inhibition of IGF-1R activity blocks signaling downstream of TSHR (46). IGF-1R/TSHR signaling also boosts the production of hyaluronan in GO OFs and other glycosaminoglycan components in the ECM (48) (**Figure 2**). Increased deposition of ECM, especially high molecular weight hyaluronan, can promote enlargement, inflammation, edema and congestion of GO orbital tissue (49). Activation of the IGF-1R/TSHR protein complex also increases the secretion IL-6 and IL-8, which are involved in the autoimmune response to GO. Teprotumumab is the only drug approved by the FDA for the treatment of GO. Teprotumumab is a human monoclonal IGF-1R blocking antibody that can achieve therapeutic effects for GO by blocking IGF-1R/TSHR crosstalk-mediated increase in hyaluronate synthesis and adipogenesis on the surface of OFs (50, 51). A recent randomized, multicenter, placebo-controlled, double-blind trial showed that treatment with Teprotumumab in GO patients was more effective than placebo in reducing ocular proptosis and CAS (52).

## 3 Non-coding RNA

Epigenetics is a research hotspot in recent years, which refers to heritable changes in gene function without changes in the DNA sequence of genes, and ultimately leads to changes in phenotype, such as histone modifications, DNA methylation, and non-coding RNA (ncRNA). Here we mainly elaborate the relationship between ncRNA and GO.

NcRNA refers to a class of RNA transcripts lacking protein-coding functions, which can be classified into microRNA (miRNA), circular RNA (circRNA) and long non-coding RNA (lncRNA). It is reported that their abnormal expression is importantly associated with the pathophysiology of autoimmune thyroid disease (53) (**Table 1**).

**Table 1:** The role of non-coding RNAs in the pathogenesis of GO.

Noncoding RNAs	Samples/cells	Expression change	Function	Effects in GO		References	
miR-146a	peripheral blood	Down-regulated	Inhibition of Th1 differentiation and cell proliferation processes in CD4+ T lymphocytes	Promotes inflammation		(54)	
	Plasma/Serum	Down-regulated	Promotes Th17 cell differentiation	Promotes inflammation		(55)	
	Orbital adipose/connective tissue	Up-regulated	Inhibition of TGF-β-induced fibrosis marker production	Inhibition of fibrosis process		(56)	
	Orbital tissue	Up-regulated	Inhibition of IL-1β-induced IL-6 protein production and ICAM-1 expression	Relief of inflammation		(57)	
miR-146a and miR-155	OFs	Up-regulated	Reduced expression of ZNRF3 and PTEN	Enhancement of OFs proliferation		(58)	
miR-Let7-5p	Serum	Down-regulated	Prevention of Th1 cell-mediated IFN-γ secretion and inflammatory response	Relief of inflammation		(59)	
miR-183 and miR96	peripheral blood	Up-regulated	Promotes proliferation of CD4+ T cells	Promotes inflammation		(60)	
miR-224-5p	Serum	Up-regulated	Increased GC sensitivity and GR expression *via* GSK-3β	Increase GC treatment sensitivity		(61)	
miR-885-3p	Plasma exosomes	Up-regulated	Inhibition of AKT/NFκB signaling pathway, up-regulated of GR levels and down-regulated of inflammatory factor levels	Increase GC treatment sensitivity and alleviate inflammatory response		(62)	
miR-27a-3p	Plasma/Serum	Up-regulated	——	As a potential biomarker for predicting the progression of GD to GO		(63)	
miR-22-3p	Plasma/Serum	Down-regulated	——	As a potential biomarker for predicting the progression of GD to GO		(63)	
miR-29	OFs	Up-regulated	Inhibition of TGF-β-mediated synthesis of the ECM of OFs	Inhibition of fibrosis process		(64)	
miR-21	Orbital adipose tissue	Up-regulated	Inhibits PDCD4 expression, thereby promoting the proliferation of OFs	Promotes the fibrosis process		(65)	
miR-27a and miR-27b	Orbital adipose tissue	Down-regulated	Inhibition of PPARG and C/EBP expression is involved in adipogenesis	Promotes adipogenesis		(66)	
miR-130a	OFs	Up-regulated	Targeting AMPK and attenuating AMPK activity to promote lipid accumulation	Excessive accumulation of adipose tissue		(67)	
circRNA_14940	Orbital adipose/connective tissue	Up-regulated	Involved in Wnt signaling pathway with up-regulated CCND1, involved in PI3K-Akt signaling pathway with down-regulated TNXB, interacts with ECM receptor	Involvement in GO pathogenesis		(68)	
circRNA_10135	Orbital adipose/connective tissue	Up-regulated	Interacts with PTGFR through calcium signaling pathway and plays a role in adipogenesis	Promotes adipogenesis		(68)	
LINCO1820:13	Orbital adipose/connective tissue	Up-regulated	RNA-induced silencing complexes that competitively bind miR-27b, thereby upregulating FPR2 expression	Involvement in GO pathogenesis	(69)			
ENST0000499452	Orbital adipose/connective tissue	Up-regulated	Competitive inhibition of miR-27a, thereby weakening its inhibitory effect on CXCL1	Involvement in GO pathogenesis	(69)			

The red values provided in Table 1 indicate the references cited in the row where each non-coding RNA is located.

### 3.1 MiRNA and GO

MiRNA is an endogenous short non-coding RNA molecule, 18 to 23 nucleotides in length, which can effectively mediate the degradation of mRNA or restrict its translation by binding to the structural domain of the 3’ untranslated region of the target gene mRNA, thereby exerting a strong negative regulatory influence on post-transcriptional levels (70). It has been shown that miRNAs play a great role in a wide range of biological activities, including immune function, apoptosis, cell differentiation and development, cell proliferation and metabolism (59). It is reported that abnormal miRNA expression has been linked to the pathogenesis of GO, and because miRNAs are easily detectable in blood and tissue samples, they are expected to be early biomarkers for evaluating disease risk and severity (71).

#### 3.1.1 MiRNA in GO circulation

It has been suggested that some circulating miRNAs have been linked to the development of GO and are closely associated with the differentiation or activation of immune cells, and the regulation of the immune response. One of the most studied miRNAs is miR-146a, which is a typical multifunctional miRNA characterized by regulatory roles in immune regulation, cell proliferation, differentiation, apoptosis and ECM (72). Several studies have found that GO patients’ peripheral blood miR-146a levels are considerably lower than healthy controls (72). Meanwhile, CAS and IL-17 levels are negatively related to down-regulated miR-146a expression, whereas IL-17 levels are positively related to CAS (55). The down-regulated of miR-146a may result in an unexpected increase in IL-17 levels, promoting the development of GO ocular inflammation. MiR-146a inhibits Th1 differentiation and cell proliferation process of CD4^+^ T lymphocytes by targeting NUMB protein (72). A recent study reported that the down-regulated miR-146a expression in CD4^+^ T lymphocytes from GO patients attenuates the above inhibitory effect, thereby promoting ocular inflammation in GO (54). In conclusion, the down-regulated circulating miR-146a expression may indirectly contribute to the development of GO by influencing the production of different inflammatory factors. The nuclear factor κB (NF-κB) gene is an important immune and inflammatory response gene. MiR-146a and miR-155 are both reported to be trans targets of NF-κB, which can constitute a negative feedback pathway through the involvement of certain genes (73). Therefore, we speculate that miR-146a and miR-155 can have the same target gene in NF-κB signaling and regulate each other’s immune response. Li et al. hypothesized that, in contrast to miR-146a, miR-155 could augment GO ocular inflammation by boosting inflammatory T cell production (74). In addition, we are speculated that miR-155 may have other functions in the pathophysiology of GO. In macrophages, miR-155 promotes the expression of TNF-α and IL-6 by targeting the SOCS1 gene (75). In systemic sclerosis, MiR-155 induces persistent collagen expression in fibroblasts and plays a critical role in fibrosis (76). In 3T3-L1 cells, however, miR-155 inhibits adipogenesis and adipocyte development (77). From this, we hypothesized that miR-155 may play an important role in GO inflammatory response, fibrosis and adipogenesis.

Rebeca et al. concluded that other miRNAs, such as miR-Let7-5p, miR-142-3p, miR-21-5p, miR-301a-3p and miR-95-5p are also differentially expressed in autoimmune thyroid disease and that in GD patients (including GO), the above miRNAs correlate with disease severity (59). When compared to GD patients who don’t have GO, miR-Let7-5p was significantly lower in GO patients. MiR-Let7-5p was negatively related to CAS, and miR-Let7-5p levels were lower in GO patients with more severe diseases (59). This is because miR-Let7 has a Treg cell-mediated activity that suppresses IFN-γ secretion and inflammatory responses by Th1 cells (59), so the low levels of miR-Let7-5p discovered in GO might be linked to the previously observed defective Treg cellular function in individuals with autoimmune thyroid disease (23). It has been reported that miR-21 has been linked to the regulation of immune function as well as the development of numerous autoimmune disorders (78, 79), from which we can speculate that miR-21 may have a common mechanism of action in different immune cells. The reality that miR-21 expression can affect T cell activation, including Th1/Th2 balance (80), and Th17 cell differentiation (81), could explain the above findings.

Thiel et al. reported that miR-96 and miR-183 were abundantly expressed in CD4^+^T cells from GO patients’ peripheral blood and in human and mouse T cells activated *in vitro* (60). They proposed that these two miRNAs boost CD4^+^ T cell proliferation by influencing the expression of EGR1 associated with the PTEN-PI3K-AKT signaling pathway, leading to their expansion *in vivo* and thus promoting the development and progression of GO.

Shen et al. reported that low levels of miR-224-5p in serum may be associated with insensitivity to glucocorticoid (GC) therapy in GO patients. MiR-224-5p may serve as a valid biomarker to predict the efficacy of GC therapy in GO patients, and they hypothesized that circulating miR-224-5p overexpression may increase GC receptor and GC sensitivity expression by targeting glycogen synthase kinase-3β (61). Sun et al. found that miR-885-3p was up-regulated in the plasma of GO patients with improved symptoms after intravenous GC treatment (62), miR-885-3p inhibits the AKT/NFκB signaling pathway and up-regulated GRE luciferase reporter gene plasmids and GC receptor levels to improve GC sensitivity and down-regulated inflammatory factor levels in OFs to alleviate GO autoimmune responses. Therefore, miR-885-3p can also be used as a biomarker to assess intravenous GC sensitivity in GO patients. Zhang et al. found by high-throughput proteomics and miRNA sequencing experiments that the up-regulated miR-27a-3p and down-regulated miR-22-3p in expression levels from GO serum/plasma compared to healthy controls, can be employed as possible biomarkers to predict the progression of GD to GO (63).

In summary, we hypothesized that detection of various miRNAs differentially expressed in GO patients could be employed as potential biomarkers to predict the progression of GO.

#### 3.1.2 MiRNA in GO orbital tissue

In addition to being expressed in the peripheral circulation, miRNAs from GO patients can also be differentially expressed in orbital tissue. OFs play a role in the pathophysiology of GO as both target and effector cells. Several recent researches have demonstrated that some miRNAs may be link to GO orbital tissue fibrosis, autoimmune response and adipose tissue formation.

It has been shown that miR-146a in orbital tissue is associated with fibrotic, inflammatory responses in GO orbital tissues. Sun et al. found significantly higher miR-146a expression levels in the orbital adipose tissue of GO patients than in non-GO orbital adipose tissue (56). And then they found experimentally that miR-146a may become connected to the production of TGF-β-induced fibrosis markers such as fibronectin, collagen type Iα and α smooth muscle actin as negative regulators, i.e. miR-146a may be regulated the antifibrosis in OFs of GO patients (56). However, it has also been reported that TSHR signaling in GO OFs can enhance the proliferation of GO OFs by inducing the production of miR-146a and miR-155 to reduce the expression of target genes that block cell proliferation ZNRF3 and PTEN (58). The pathological process of partial fibrous hyperplasia reported in GO could be explained by the TSHR-dependent expression of miR-146a and miR-155. However, Sun et al. discovered that miR-146a is also connected to the anti-inflammatory process of GO in prior work. By analyzing miR-146a mimics, they discovered that miR-146a mimics inhibited IL-1β-induced IL-6 protein synthesis and intercellular adhesion molecule-1 expression (57). This finding supports a previous study that found miR-146a inhibits the NF-κB pathway by downregulating its target genes, such as IRAK1 and TRAF6 (82), resulting in the relief or termination of the inflammatory response. Comparing the relationship between miR-146a and inflammatory response in peripheral blood, we can conclude that different levels of miR-146a play different roles in the inflammatory response of OFs by regulating the NF-κB pathway. The down-regulated of miR-146a levels promotes the development of inflammation, while the up-regulated of its levels allow the remission or termination of the inflammatory response. It has been proposed that miR-155 plays a crucial role in the development of Th17 cells during autoimmunity (69). Also, miR-155 has an essential role in fibrosis by mediating TGF-1β1 signaling to drive collagen synthesis (83). However, the precise mechanism of miR-155 in GO is yet unknown, and more research is needed.

In addition to miR-146a, miR-29 and miR-21 have also been reported to be associated with OFs fibrosis. Similar to miR-146a, miR-29 overexpression significantly inhibited TGF-β-mediated synthesis of the ECM of OFs (64). In contrast to miR-146a, PDGF-BB significantly inhibits PDCD4 expression by upregulating miR-21 in OFs, thereby promoting the proliferation of OFs (65).

MiR-27a and miR-27b inhibit adipose differentiation in GO patients’ OFs. Sun et al. discovered that GO patients’ orbital adipose tissue had considerably lower levels of miR-27a and miR-27b than non-GO tissue by real-time enzyme chain polymerization reaction, and reduced levels of adipogenesis-induced PPARG, C/EBP-α and C/EBP-β proteins and miRNA expression in miR-27a and miR-27b mock-transfected OFs (66). These findings imply that down-regulated miR-27a and miR-27b expression may be implicated in adipocyte formation by inhibiting PPARG and C/EBP expression (66). We thus speculate that miR-27a and miR-27b, *via* modulating the formation of adipocytes, may provide potential therapeutic targets for the treatment of GO-induced ocular proptosis. In addition to miR-27a and miR-27b, miR-130a also plays a role in GO orbital tissue adipose tissue formation. Hammond et al. first found that miR-130a was elevated in patients with OFs prone to lipogenesis and promoted lipid accumulation, and that miR-130a also targeted and attenuated adenosine monophosphate-activated protein kinase activity and promoted lipid accumulation (67). This new mechanism may lead to new treatments for patients with GO.

In summary, miRNAs play a crucial role in the development of GO, with miR-146a being a promising target. However, we do not fully understand the mechanism of miRNA action in GO, so additional research is needed to elucidate the application of miRNA in GO.

### 3.2 CircRNA and GO

CircRNA is an RNA molecule with a closed-loop structure, which is a characteristic structure generated by covalent bonds at the 3’ and 5’ ends after reverse splicing (84). Based on the biogenesis pattern of genomic regions, circRNAs can be classified into four categories: intronic circular RNA, exonic circular RNA, exon-intron circular RNA, and intergenic circular RNA (85). CircRNAs provide a variety of biological functions, including serving as miRNA sponges for adsorption, attachment to various RNA-binding proteins, and engagement in protein translation (86).

But current research on the role of circRNAs in the pathophysiology of GO is in its early stages. Wu et al. derived from RNA sequencing of orbital adipose/connective tissue samples from GO patients that 1631 circRNAs were differentially expressed in GO samples (68). Analysis of circRNA-miRNA co-expression and circRNA-miRNA interactions showed that circRNA_14940 may be involved in GO pathogenesis by participating in the Wnt signaling pathway with up-regulated CCND1, interaction with down-regulated TNXB involved in ECM receptor, focal adhesion and PI3K-AKT signaling pathway (68). Therefore, differentially expressed circRNAs may have a role in GO pathogenesis, with the circRNA_14940-CCND1-Wnt signaling pathway being a key regulatory axis. Wu et al. hypothesized that circRNA_10135 may interact with the up-regulated PTGFR through the calcium signaling pathway and play a role in adipogenesis in GO (68). In addition, they hypothesized that hsa-miR-10392-3p may function in GO by regulating circRNA_14936, which is associated with TNFRSF19 and thus affects cytokine-cytokine receptor interactions associated with B-cell survival and, in turn, the development of GO (68). Therefore, circRNA may play a key role in the development of GO.

However, there have been few studies on the role of circRNAs in the pathogenesis of GO, and there are many circRNAs whose functional analysis is not comprehensive, so more experiments are needed for functional validation.

### 3.3 LncRNA and GO

LncRNAs are a type of long-stranded multifunctional RNAs with a length of more than 200 that have been reported to act at many different levels of gene expression, including epigenetic regulation, transcriptional regulation, post-transcriptional regulation, and miRNA regulation (87). Salmena et al. suggested the competitive endogenous RNA hypothesis for lncRNA-miRNA interactions (88), which is characterized as mutual interference between coding and non-coding RNAs *via* miRNA response elements, resulting in an extensive regulatory network in the transcriptome (69).

Yue et al. derived by quantitative real-time PCR that there were differences in the expression of four mRNAs (CHRM3, THBS2, FPR2, CXCL1) and two lncRNAs (LINC01820:13, ENS0000499452) between GO patients and control patients (69). They hypothesized that LINCO1820:13 up-regulated FPR2 expression by competitively binding to the RNA-induced silencing complex of miR-27b, resulting in autoimmune responses and inflammation in GO patients (69). ENST0000499452 suppresses miR-27a through the same mechanism, thereby impairing its inhibitory effect on CXCL1 and enhancing immune response, inflammation, and fibrosis in GO patients’ OFs (69).

Wu et al. found that co-expression interactions between differentially expressed lncRNAs and 52 ECM-related mRNAs (e.g., COL12A1, TNXB, COL6A3, KAZALD1, FBN1 and SPON1) in GO patients’ orbital adipose/connective tissue samples suggest that lncRNAs may regulate ECM function in GO patients’ orbital adipose/connective tissue from (89). For example, co-expression interactions of lncRNA with COL12A1 and negative co-expression of lnc-PTP4A2-3:7 with TNXB may be associated with altered ECM in GO orbital adipose/connective tissue (89). Wang et al. shown that the combination of upregulated lncRNA LPAL2 and downregulated miR-1287-5p in orbital tissues of GO patients induced increased levels of cell adhesion factors and activation of GO OFs by TGF-β1 through EGFR/AKT signaling (90). Meanwhile, EGFR has been reported to increase the proliferation of OFs, which may contribute to the fibrosis of extraocular muscles (91).

In conclusion, lncRNAs have a significant role in the pathophysiology of GO, and we need to conduct more studies in the future to find the relationship between lncRNAs and the pathogenesis of GO.

## 4 Exosomes and GO

**Figure 3:**
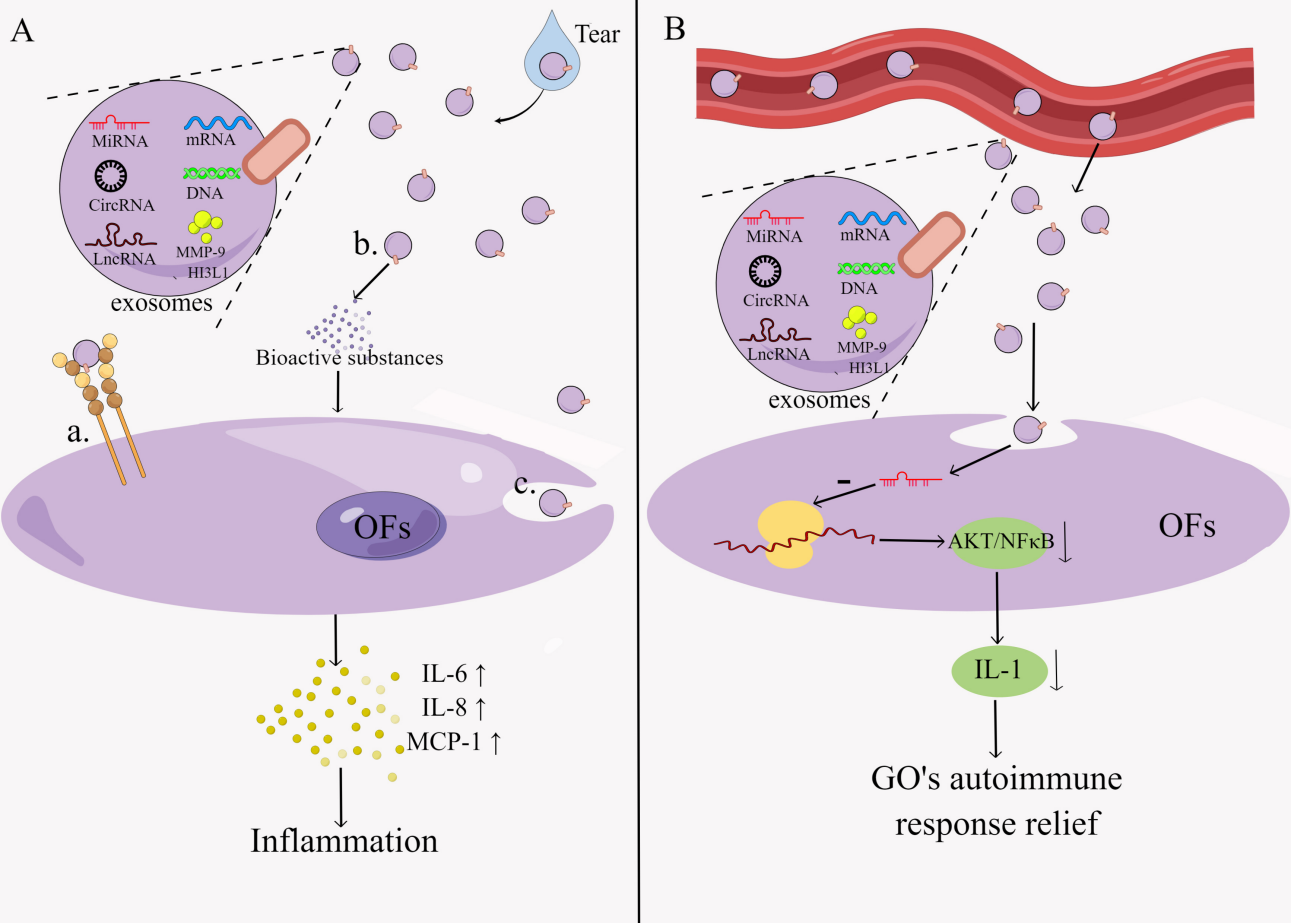
Pathogenesis of exosomes in GO.Exosomes regulate the immune response by two main mechanisms: **(A)** exosomes act directly on target cells thereby activating downstream signals. This process is characterized by: a. The direct action of surface signaling molecules. b. Extracellular release of bioactive substances. c. Intracellular regulation of signaling molecules during membrane fusion. **(B)** Exosomes mediate regulation of immune responses through exosomal miRNA. By Figdraw (www.figdraw.com).

Exosomes are extracellular vesicles that are 30-150 nm in size and can transport specific compounds such as proteins, lipids, miRNA, DNA, etc (92). It can be found in many different body fluids, including blood, tears urine, breast milk, saliva, etc. (93). Exosomes contain a wide range of bioactive molecules, including chemokines, inflammatory factors, signal transduction factors, different RNAs, etc., and proteins with specialized roles on their surface, such as adhesion molecules, co-stimulatory molecules, ligands, receptors, and so on (92). Exosomes regulate the immunological response through two main mechanisms: direct action of exosomes on target cells to activate downstream signals and exosomal regulation of the immune response mediated by exosomal miRNAs (94) (**Figure 3**). The former works in three ways: surface signaling molecules act directly, bioactive substances are released extracellularly, and signaling molecules are modulated intracellularly during membrane fusion (95). The immunomodulatory effects of exosomes include T cell activation, antigen presentation, inflammatory response, immunosuppression and intercellular communication (96). Exosomes have been implicated in the initiation and progression of GO in several studies, but research into them is still in its early stages.

Han et al. demonstrated that exosomes in tears of GO patients were 2.3-fold higher than in healthy controls and that exosomes triggered the release of inflammatory cytokines IL-6, IL-8 and monocyte repellent protein-1 from OFs in an *in vitro* experiment. These data imply that the abundance of specific proteins (e.g., MMP-9, CHI3L1) in exosomes can activate the inflammatory response, orbital tissue remodeling, and fibrosis in OFs of GO patients (97). MMP are enzymes that play a key role in fibrosis, inflammatory processes and tissue remodeling, and previous reports have shown increased serum MMP-9 concentrations in GO patients and an association with CAS in GO patients (98). CHI3L1 increases the synthesis of inflammatory cytokines like IL-1β, IL-6, TNF-α, and IFN-γ, connective tissue growth and fibrosis (99).

Sun et al. discovered that differences in miRNA levels in GO patients were caused by changes in miRNA content in exosomes rather than changes in exosome concentrations per unit volume of plasma, and that exosome-delivered miR-885-3p could inhibit the AKT/NFκB signaling pathway, affecting OFs in GO patients (62). This suggests that plasma exosomes’ miRNAs can be transferred to recipient cells *via* exosomes and bind to target cells, which then regulate cellular functions.

Based on the current evidence, exosomes have great potential as diagnostic biomarkers and therapeutic approaches for GO. However, exosome research is still in its infancy, and their potential mechanisms in GO have yet to be fully elucidated. Therefore, we need to continue our research to determine the specific mechanism of exosomes in GO, which will contribute to a better understanding of the diagnosis and prognosis of the disease.

## 5 Conclusion and prospect

GO is an autoimmune disease with multiple factors involved in its pathogenesis, and its pathogenesis is still being explored. Cellular immunity plays a key role in orbital inflammation in GO, where T cells interact with OFs through the production of various cytokines, as well as induce the propagation of multiple intracellular signaling cascades, resulting in hyaluronan secretion, adipogenesis, and the persistence of orbital inflammation. TSHR, one of the major autoantigens of GO, is associated with immune response and adipogenesis in orbital tissues, and also induces IL-6 expression and secretion, thereby exacerbating the autoimmune response. IGF-1R on OFs is involved in adipogenesis and hyaluronan synthesis, as well as mediating some aspects of orbital changes. The researchers found that IGF-1R and TSHR can form physical and functional protein complexes that are involved in the development of GO. With the advent of high-throughput gene sequencing technology, the importance of ncRNA in the pathogenesis of GO has been gradually revealed, and current studies have found that it is mainly related to cell differentiation, immune regulation, and adipogenesis. Exosomes have received a lot of attention in recent years, mainly through direct action on target cells to trigger downstream signals and through exosomal miRNAs to regulate GO immune responses, orbital tissue remodeling and fibrosis. However, these studies are still in the exploratory stage, and revealing the molecular mechanisms behind GO is expected to provide insights for formulating new treatment plans, developing new therapeutic strategies, and optimizing our clinical management of the disease.

Currently, we most commonly use GC for the treatment of GO, which have immunosuppressive and anti-inflammatory effects and can be used to alleviate the clinical symptoms of GO. However, long-term or high-dose GC treatment can lead to many complications, such as medically induced Cushing’s syndrome, diabetes, hypertension and osteoporosis, etc. Unfortunately, a small percentage of GO patients are resistant to GC treatment, making treatment more challenging. Some biologics developed for immune mechanisms, such as Infliximab, Etanercept, Tocilizumab and Teprotumumab, have shown promising results in the treatment of GO, but because their drugs are expensive and require multiple intravenous treatments, they can impose a significant financial burden on patients. The current treatment methods are difficult to meet the patient’s treatment requirements. Therefore, it is imperative to conduct more effective clinical studies to explore more effective and safer drugs for the long-term treatment of GO.

## Author contributions

JZ, HD and HH wrote the manuscript. JZ and HD wrote the first draft of the manuscript. HH revised the manuscript and edited. SY, BL and YC assisted in manuscript preparation. All authors contributed to the article and approved the submitted version.

## Funding

This research was supported by the Second Affiliated Hospital of Fujian Medical University horizontal scientific research project (NO: HX202201 and HX202202), the Second Affiliated Hospital of Fujian Medical University Doctoral Research Project (NO: 2021GCC03).

## Acknowledgments

Thanks to Figdraw (www.figdraw.com) for their support in drawing the figure.

## Conflict of interest

The authors declare that the research was conducted in the absence of any commercial or financial relationships that could be construed as a potential conflict of interest.

## Publisher’s note

All claims expressed in this article are solely those of the authors and do not necessarily represent those of their affiliated organizations, or those of the publisher, the editors and the reviewers. Any product that may be evaluated in this article, or claim that may be made by its manufacturer, is not guaranteed or endorsed by the publisher.
